# Autoimmmune hepatitis

**DOI:** 10.1038/s41423-021-00768-8

**Published:** 2021-09-27

**Authors:** Benedetta Terziroli Beretta-Piccoli, Giorgina Mieli-Vergani, Diego Vergani

**Affiliations:** 1 Epatocentro Ticino & Facoltà di Scienze Biomediche, Università della Svizzera Italiana, Lugano, Switzerland; 2 Institute for Research in Biomedicine, Bellinzona, Switzerland; 3 King’s College London Faculty of Life Sciences & Medicine at King’s College Hospital, London, UK; 4 Paediatric Liver, GI and Nutrition Centre, MowatLabs, King’s College Hospital, London, UK; 5 Institute of Liver Studies, MowatLabs, King’s College Hospital, London, UK

**Keywords:** Autoimmune Hepatitis, Immunopathophysiology, Treatment, Genetic Predisposition, Autoimmunity, Autoimmune diseases

## Abstract

Autoimmune hepatitis (AIH) is a T-cell mediated, inflammatory liver disease affecting all ages and characterized by female preponderance, elevated serum transaminase and immunoglobulin G levels, positive circulating autoantibodies, and presence of interface hepatitis at liver histology. AIH type 1, affecting both adults and children, is defined by positive anti-nuclear and/or anti-smooth muscle antibodies, while type 2 AIH, affecting mostly children, is defined by positive anti-liver-kidney microsomal type 1 and/or anti-liver cytosol type 1 antibody. While the autoantigens of type 2 AIH are well defined, being the cytochrome P4502D6 (CYP2D6) and the formiminotransferase cyclodeaminase (FTCD), in type 1 AIH they remain to be identified. AIH-1 predisposition is conferred by possession of the MHC class II HLA DRB1*03 at all ages, while DRB1*04 predisposes to late onset disease; AIH-2 is associated with possession of DRB1*07 and DRB1*03. The majority of patients responds well to standard immunosuppressive treatment, based on steroid and azathioprine; second- and third-line drugs should be considered in case of intolerance or insufficient response. This review offers a comprehensive overview of pathophysiological and clinical aspects of AIH.

## Introduction

Autoimmune hepatitis (AIH) is a chronic inflammatory condition of the liver due to an autoimmune attack against hepatocytes. What triggers the disease remains unknown, though risk factors have been reported, and, particularly in type 2 AIH, target autoantigens have been identified [1]. AIH is characterized clinically by female preponderance and variable presentation, biochemically by high serum levels of transaminases, serologically by elevated immunoglobulin G (IgG) and positive circulating autoantibodies, and histologically by interface hepatitis. It affects all ages, including young children. AIH is subdivided into two types according to the serological profile: type 1 (AIH-1) is characterized by anti-nuclear antibody (ANA) and/or anti-smooth muscle antibody (SMA), whereas type 2 (AIH-2) is characterized by anti-liver-kidney microsomal antibody type 1 (anti-LKM1) and/or by anti-liver cytosol type 1 antibody (anti-LC1) [2].

AIH-1 patients may have cholestatic features meeting the diagnostic criteria either of primary biliary cholangitis (PBC), i.e. positive anti-mitochondrial antibody, biochemical cholestasis and non-suppurative destructive cholangitis at liver histology, or of primary/autoimmune sclerosing cholangitis (PSC/ASC), i.e. abnormal cholangiogram [3–6] (Table 1).

**Table 1:** Overview of the main clinical features of autoimmune liver diseases

	Sex distribution	Typical age at onset	Autoantibodies	Liver histology hallmark	Commonly associated extrahepatic conditions	First-line treatment
Autoimmune hepatitis	F > M	Childhood/adolescence; middle-age	ANA, SMA, anti-SLA, anti-LKM1, anti-LC1	Interface hepatitis	Autoimmune thyroid disease; type 1 diabetes; IgA deficiency	Corticosteroid ± azathioprine
Primary biliary cholangitis	F ≫ M	>45 years	AMA, ANA with rim-like or multiple nuclear dots patterns	Non-suppurative destructive cholangitis	Autoimmune thyroid disease; Sjögren syndrome	UDCA
Primary sclerosing cholangitis	M > F	25–40 years	pANNA	Portal inflammation, ductular proliferation	Inflammatory bowel disease	UDCA*, liver transplantation
Autoimmune sclerosing cholangitis	F = M	Mainly described in childhood	ANA, SMA, anti-SLA pANNA	Interface hepatitis and cholangiolitis	Inflammatory bowel disease; autoimmune thyroid disease; type 1 diabetes	Corticosteroid ± azathioprine and UDCA
Primary biliary cholangitis variant syndrome	F > M	>45 years	ANA, SMA + AMA	Non-suppurative destructive cholangitis and interface hepatitis	Autoimmune thyroid disease; Sjögren syndrome	corticosteroid ± azathioprine and UDCA
Primary sclerosing cholangitis variant syndrome	Unknown	Young adulthood	ANA, SMA, anti-SLA pANNA	Interface hepatitis and cholangiolitis	Inflammatory bowel disease	corticosteroid ± azathioprine and UDCA*

*F* female, *M* male, *ANA* anti-nuclear antibody, *SMA* anti-smooth muscle antibody, *SLA* soluble liver antigen, *LKM1* liver kidney microsomal type 1, *LC1* liver cytosol type 1, *AMA* anti-mitochondrial antibody, *pANNA* peripheral anti-nuclear neutrophil antibody, *AIH* autoimmune hepatitis, *PBC* primary biliary cholangitis, *PSC* primary sclerosing cholangitis, *UDCA* ursodeoxycholic acid

*Unclear evidence of long-term benefit

### Historical notes

The first observation of AIH dates back to the 1940s, when a chronic hepatitis with high serum proteins and female preponderance was noted [4]. The disease was better characterized a few years later by the Swedish physician Waldenström, who presented at the meeting of the German Society for Digestive and Metabolic Disorders in 1950 his observations on six patients, five females, affected by a peculiar form of hepatitis (‘hepatitis sui generis’) with marked elevation of serum gamma globulins and amenorrhea, who had a striking improvement of symptoms and a dramatic fall of the erythrocyte sedimentation rate after administration of adrenocorticotropic hormone [5]. At that time, liver biopsy, serum transaminase levels and autoantibodies were not used in clinical practice [4]. The condition was called chronic active hepatitis. The first hint of an autoimmune origin of the disease was the observation of lupus erythematosus cells in blood and ascites of patients with hypergammaglobulinemic hepatitis [4]. In a landmark paper published in the Lancet in 1956 by Ian Mackay, who can be considered the father of AIH, five additional cases were reported, and the condition was defined as ‘lupoid hepatitis’ [6]. This name was later abandoned and replaced by AIH, as it became clear that lupus erythematosus is a distinct clinical entity, rarely coexisting with AIH in the same patient [7]. The introduction of indirect immunofluorescence (IIF) led to the discovery of SMA, which was often present in AIH, but not in lupus erythematosus, helping in the differentiation of the two diseases.

AIH-2 was first reported in 1987 by Homberg et al. in children with an aggressive form of chronic active hepatitis, positive for anti-LKM1 but negative for ANA and SMA [8].

After the identification of several possible causes of chronic hepatitis, diagnostic criteria for AIH were published by the International Autoimmune Hepatitis Group (IAIHG) in 1993 [9].

### Epidemiology

AIH conforms to the definition of a rare disease, affecting less than 200,000 individuals in the US and less than 1 in 2000 inhabitants in the EU. It occurs worldwide and in all ethnicities, but the vast majority of the epidemiological studies stem from Western countries, and, more recently, from Asia [1, 10].

Early epidemiological studies, carried out before the publication of diagnostic criteria, report an incidence ranging from 0.1 to 1.9 cases/100,000 in European countries and Japan [1]. More recent studies from Europe report higher disease frequency, with an incidence ranging from 1.1 to 2.56 and a prevalence ranging from 17.3 to 18.3/100,000 inhabitants [11–14]. Studies in more recent years report an even higher incidence [11, 14].

A large primary care population-based study from the UK published recently reported a yearly incidence of AIH of 1.94/100,000 inhabitants from 2002 to 2016 [15]: the authors describe a higher incidence with higher latitude; in contrast to previous studies, this report did not find an increasing incidence in more recent years. The median age at disease onset in this study, which included only adults, increased from 2002 to 2015 from 52 to 58 years.

A recent study from the US, based on a commercial database including some 37,000,000 patients of all ages from 2014 to 2019, reported an AIH prevalence as high as 31.2/100,000 [16].

A population-based study from New Zealand published in 2021 reported an overall incidence of 1.93/100,000 from 2008 to 2016, with a significant increase of the incidence from the first 3 years of the observation period to the last three years (1.37–2.39). This is in line with recent European studies [13, 14], and probably reflects a true increase in the disease frequency, as observed for other autoimmune diseases. The point prevalence in 2016 in the study from New Zealand was 27.4/100,000 [17]. The highest disease prevalence has been reported in Alaska (42.9/100,000) [18].

AIH has long being considered to be rarer in Asia, although a recent systematic review of the literature published until April 2019 reported a similar yearly incidence per 100,000 inhabitants in Asia (1.31), Europe (1.37) and in America (1.00) [10]. The worldwide AIH prevalence in this paper was 17.44/100,000 inhabitants, being lower in Asia as compared to Europe and America [10]. This finding may reflect a more recent awareness of AIH in Asia.

The epidemiology of AIH-2 has been less investigated, although it is well known that it is much rarer than AIH-1 and affects mostly children. In a recent study from Argentina including 56 children/adolescents, the yearly incidence of pediatric AIH from 2003 to 2013 was 0.56/100,000 inhabitants aged 0–18 years, only 11% of the incident cases being AIH-2 [19]. In a Canadian study of 159 children/adolescents, the annual incidence was 0.23/100,000 children, type 1 AIH being 5.5 times more frequent than type 2 AIH [20]. The real incidence of AIH-2, however, remains to be established as the defining autoantibodies are not universally tested [21].

### Risk factors and pathophysiology

The etiology of AIH remains unknown, while pathophysiological mechanisms and risk factors have been described and are constantly updated.

### Risk factors

Female sex is a clear risk factor for AIH: in all populations, three quarters of AIH patients are female [22]. This feature is shared with the majority of autoimmune diseases, but not with ASC and PSC. AIH was first reported in young women, leading to consider it a disease of this age group, but from the late 90 s AIH onset after the age of 60 has increasingly been reported in several populations and geographic areas including Europe, North America, China, India and New Zealand [17, 23–27]: therefore, AIH should always be suspected also in elderly patients with acute or chronic liver disease. AIH patients diagnosed after the age of 60 have more often cirrhosis, suggesting long-standing unrecognized disease [28].

Viral infections have been reported to be a risk factor for AIH, providing an insight in the pathogenetic mechanism of molecular mimicry, whereby immune responses to pathogens are redirected towards structurally similar self-antigens [29]. This has been best described in AIH-2, in which an amino acid sequence of the target autoantigen CYP2D6 is shared in common with sequences of hepatitis C virus proteins, and other viruses belonging to the herpesvirus family [1]. Moreover, anti-LKM1 is detected in up to 10% of HCV infected subjects, with a tendency to disappear after HCV clearance [30]. Exposure to drugs, particularly nitrofurantoin, minocycline, anti-tumor necrosis factor alpha (TNFα) and statins, but also to herbal supplements, in predisposed subjects, may cause drug-induced liver disease (DILI) mimicking AIH (see below) [31].

Genetic predisposition is conferred mainly by polymorphisms in human leukocyte antigen (HLA) alleles, as shown by early studies and later confirmed by a genome-wide association European study [32, 33]. However, this is not sufficient to trigger the disease since HLA predisposing to AIH are found in up to 30% of the healthy general Caucasian population.

In adults, susceptibility to AIH-1 has been linked to MHC class II HLA DRB1 alleles encoding the similar amino acid sequences LLEQKR and LLEQRR at positions 67–72 of the DRβ polypeptide. These motifs are encoded by the DRB1*0301 and DRB1*0401 alleles, which predispose Caucasian adults from Northern Europe, Northern America and Iran to AIH-1 [34–36]; by DRB1*0405, allele imparting susceptibility in Japan and Argentina [37, 38]; and by DRB1*0404, reported to be the AIH-1 predisposing allele in Mexico [39], though this has been later questioned [40]. A recent paper suggests the impact of specific killer cell immunoglobulin-like receptors (KIR)/HLA pairs in conferring susceptibility and influencing disease progression in Japanese patients with AIH-1 [41]. DRB1*1501, which is associated with protection towards AIH-1, encodes alanine at position 71, suggesting that the amino acid at this position is a primary determinant of disease susceptibility or resistance [42–45].

Reports of MHC-encoded disease susceptibility in pediatric autoimmune liver disease have been limited until recently to either small numbers of patients or AIH subgroups [34, 46–50] and have not differentiated AIH-1 from ASC. In northern Europe, pediatric AIH-1, similar to adult AIH, is associated with the possession of HLA DRB1*03 [51, 52]. In contrast to adult patients, DRB1*04 does not predispose to AIH in childhood and can even exert a protective role [52]. AIH-2 is associated with DRB1*07, and, in DR7 negative patients, with DRB1*03 [53, 54]. In Egypt AIH-2 has also been associated with HLA-DRB1*15 [55]. In Brazil and in Egypt, the primary susceptibility allele for juvenile AIH-1 is DRB1*1301, but a secondary association with DRB1*0301 has also been identified [55, 56]. Interestingly, in South America (Argentina and Venezuela), possession of the HLA DRB1*1301 allele, which does not conform to the shared motif model mentioned above, harbouring the sequence LIEDER at positions 67–72 [44, 45, 57], not only does predispose to pediatric AIH-1 but is also associated with persistent infection with the endemic hepatitis A virus [43, 58].

Pediatric patients with AIH, whether type 1 or 2, have isolated partial deficiency of the HLA class III complement component C4, which is genetically determined [59]. AIH-2 can be part of the autoimmune polyendocrinopathy-candidiasis-ectodermal dystrophy (APECED) syndrome, in which the liver disease is reportedly present in some 20–30% of cases [60, 61].

In a recent series of 232 children of European ancestry with autoimmune liver disease carefully phenotyped from presentation and followed up for four decades, the authors define both HLA class I and II profiles for each subgroup of childhood autoimmune liver disease: DRB1*03 for AIH-1, DRB1*03 plus DRB1*07 for AIH-2 and DRB1*13 for ASC. DRB1*03 and the A1-B8-DR3 haplotype are disease predisposing genes for all three subgroups. The influence of HLA class II genes on disease severity is strong, DRB1*03 homozygosity and possession of DRB1*13 being associated to histologically more advanced disease from onset, while DRB1*07 is linked to the least optimal response to immunosuppression [62].

Less strong AIH predisposition conferred by non-HLA genetic polymorphisms has also been reported [32].

### Pathophysiology

AIH is characterized histologically by a dense infiltrate of lymphocytes, macrophages and plasma cells in the liver (see below). Despite the presence of circulating autoantibodies and plasma cell liver infiltration, AIH is considered a T cell disease, since B cell activation is a T cell dependent event [63, 64]. The key pathogenic role of T cells in AIH is mirrored by the disease predisposition conferred by HLA class II polymorphisms.

**Fig. 1:**
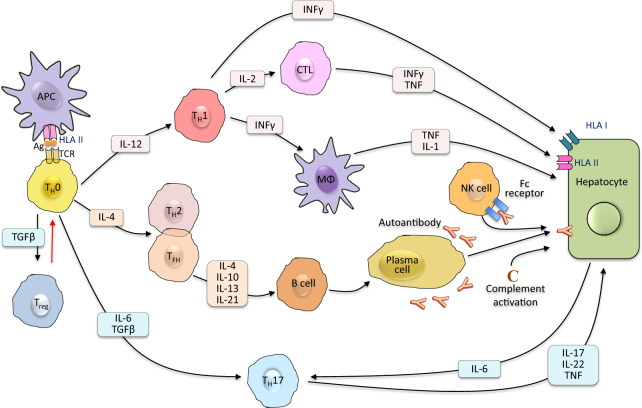
Autoimmune attack to the liver cell. An autoantigenic peptide (Ag) is presented to the T cell receptor (TCR) of uncommitted T helper (Th0) lymphocytes within the HLA class II (HLA II) molecule of an antigen-presenting cell (APC) either in the regional lymph nodes or within the liver itself. Activated Th0 cells differentiate into Th1 or Th2 cells in the presence of interleukin (IL)-12 or IL-4, respectively, and according to the nature of the antigen. This triggers a cascade of immune reactions determined by the cytokines they produce. Th1 cells secrete IL-2 and interferon (IFN)-γ, cytokines that stimulate cytotoxic T lymphocytes (CTL), enhance expression of class I HLA (HLA I) molecules, induce expression of class II HLA molecules on the liver cells and activate macrophages (MΦ). MΦ release IL-1 and tumour necrosis factor (TNF). Th2 cells secrete mainly IL-4, IL-13 and IL-21, and stimulate autoantibody production by B lymphocytes, which mature into plasma cells. Regulatory T cells (Tregs) derive from Th0 in the presence of transforming growth factor (TGF)-β. If Tregs are defective in number and/or function, hepatocyte destruction follows from the engagement of damaging effector mechanisms, including CTL, cytokines released by Th1 and by activated MΦ, complement activation, or adhesion of natural killer (NK) cells to autoantibody-coated hepatocytes through their Fc receptors. Th17 cells produce the inflammatory cytokines IL-17, IL-22 and TNF, and derive from Th0 cells in the presence of TGF-β and IL-6. The hepatocyte releases IL-6 which further stimulates Th17. Positive signal: Negative signal

Putative mechanisms of autoimmune liver damage are shown in Fig. 1. The immune response in AIH is believed to be initiated by the presentation of self-antigenic peptides, as yet unknown, to the T cell receptor (TCR) of uncommitted naive CD4 T-helper (Th0) lymphocytes. Self-antigens of interest are CYP2D6 and FTCD in AIH-2 and human SepSecS-tRNASec complex (SEPSECS) in AIH-1, as the formers are the targets of anti-LKM1 and anti-LC1, while the latter is the target of anti-soluble liver antigen (anti-SLA) (see below).

The AIH inflammatory liver infiltrate is composed mainly of α/β T cells, CD4 being twice as frequent as CD8 T cells [65]. These observations were made three decades ago and the AIH liver infiltrate should be reassessed exploiting the current advances in knowledge and technology. T cells expressing CD25, the alpha chain of the interleukin 2 (IL-2) receptor, were reported to be significantly more frequent in the liver of AIH patients as compared to patients with non-AIH liver disease [65] and were originally considered to represent exclusively effector T cells. However, in addition to the alpha chain, the IL-2 receptor contains also a beta and gamma chain and, in its trimeric form, is expressed transiently on activated T lymphocytes and constitutively on regulatory T cells. To what extent the CD25 T cells in the liver of patients with AIH represent effector or regulatory T cells remains to be fully established, and the field is in need of reassessment.

As mentioned above, the immunological mechanisms underlying naïve T cell activation and leading to the autoimmune attack to hepatocytes in AIH are initiated by presentation of autoantigenic peptides by antigen presenting cells (APC) to naïve T cells, in the presence of appropriate co-stimulation by ligand–ligand (CD28 on Th0, CD80 on APC) interaction between the two cells [1]. Self-antigenic peptides are processed and presented by professional APC, including dendritic cells (DC), macrophages, and B lymphocytes. This process takes place also in the liver, which is home to several types of APC, including Kupffer cells, liver sinusoidal endothelial cells (LSEC), DC, hepatic stellate cells and hepatocytes themselves, where antigen presentation to both CD4 and CD8 effector T cells can occur in situ, perhaps avoiding the need for trafficking to the regional lymphoid tissues [66].

The classical view holds that upon antigen priming, naïve T cells differentiate into distinct T helper cell populations, depending on the antigen and the cytokine milieu to which they are exposed [67]. In the presence of IL-12, naïve T cells differentiate into Th1 cells, whereas in the presence of IL-4 differentiation toward Th2 cells is favored, promoting B cell activation and autoantibody production. If the cytokine milieu contains an abundance of IL-1β, IL-6 and transforming growth factor-β (TGFβ), naïve T cells differentiate into Th17 cells. At variance with the view that the activation of one T cell loop suppresses the activation of the others, in AIH, a pathogenic involvement of all the three main Th subsets (Th1, Th2 and Th17) has been reported, although the role of Th17 is less clear [1, 68]. The engagement of naïve T cells into the Th1 differentiation loop leads to production of IL-2, interferon γ (IFNγ) and TNFα, as well as activation of cytotoxic CD8 T cells, which, upon interaction with HLA class I-antigenic peptide complexes on hepatocytes, cause hepatocellular damage [69, 70]. IFNγ promotes aberrant expression of HLA class II antigens on hepatocytes, rendering them able to present antigenic peptides, thus enhancing T cell activation [1, 66]. The number of peripheral blood CD8 T cells producing IFNγ in AIH-2 is significantly higher at diagnosis than during effective immunosuppressive treatment [71]. Th1 cells producing TNFα have been reported to be highly represented in the liver inflammatory infiltrates of AIH-1 patients, and IFNγ-producing Th1 cells have been reported in AIH-2 liver inflammatory cell infiltrate [70, 72]. Moreover, in a Chinese study, peripheral blood mononuclear cells (PBMC) of AIH-1 patients were found to produce high amounts of IFNγ upon incubation with peptides spanning the SEPSECS protein, potentially a key antigenic target in this condition [73].

The involvement of the Th2 cell differentiation loop in AIH is attested by the presence of plasma cells in the damaged liver and of circulating autoantibodies, a key feature of AIH, where they act as diagnostic and classification markers. Notably, autoantibodies can inflict damage themselves by antibody mediated cytotoxicity and by complement activation [74]. In this context of note is that CYP2D6 is expressed on the hepatocyte cell membrane, and is therefore accessible to anti-LKM1 [75]. Anti-LKM1, anti-LC1 and SMA serum titers correlate with disease activity, indicating their potential pathogenic role [2]. Beside a preponderant Th1 response, as detected by release of IFNγ, PBMC from AIH-2 patients stimulated with CYP2D6 peptides can also produce IL-4, indicating a Th2 engagement [53]. Interestingly, few peptides are able to trigger both pathways [53].

The involvement of Th 17, which secrete the proinflammatory cytokines IL-17, IL-22 and TNFα and promote IL-6 secretion by hepatocytes, has been investigated more recently. In a Chinese study, the frequency of IL-17-positive cells, identified by immunohistochemistry, was higher in the liver inflammatory infiltrate of AIH as compared to chronic hepatitis B (CHB) patients, both groups having mild biochemical and histological disease activity; other inflammatory liver disease controls were not investigated [76]. In the same study, serum IL-17 levels were higher in AIH as compared to healthy subjects and CHB patients [76]. A recent study from Iran reported higher IL-17 mRNA expression in PBMC of 24 untreated AIH as compared to healthy controls, but no pathological controls with inflammatory liver disease were included, questioning the relevance of the results for AIH [77]. A possible involvement of Th17 in AIH is also suggested by the fact that conversion of Tregs into Th17, IL-17 levels, and expression of RORC2—the Th17 master transcription factor—are correlated with disease activity [68, 78]. In a recent Mexican study on 46 AIH patients, serum levels of IL-17A and IL-22, which among other members of the IL-17 cytokine family is a key cytokine produced by Th17 cells, were similar in AIH and healthy controls, while levels of IL-17F were elevated in AIH, correlating with serum transaminase levels [79]. Moreover, IL-22 levels were higher in AIH-2 than in AIH-1, but only 5 AIH-2 patients were studied [79]. However, the lack of pathological controls with inflammatory liver disease different from AIH weakens the link between Th17 and AIH [79].

Emerging data suggest that follicular T helper (TFH) cells, also known as follicular B helper T cells, are involved in the pathogenesis of AIH [80]. TFH, a subset of antigen-experienced CD4 T cells, are located in secondary lymphoid organs and promote the differentiation of B cells into memory B cells and plasma cells; they secrete high amounts of IL-21 and have counterparts in the circulation. Chemokine C-C receptor 7 negative/programmed cell death-1 positive (CCR7-/PD-1 + ) TFH have been reported to be more frequent in the peripheral blood of AIH patients than in controls, including healthy subjects and patients with CHB, and have been suggested to be a diagnostic marker of AIH [81–83]. However, when appropriate controls were investigated, no difference was observed between AIH and PBC, undermining the concept that an elevation of these cells is a marker specific for AIH [83].

Three groups have recently published observations relevant to the pathogenesis of AIH. Renand et al. describe a rare population of CD4 cells, with a memory PD-1 + CXCR5 − CCR6 − CD27 + phenotype, reactive with SLA and only present in patients positive for anti-SLA autoantibodies [84]. They also describe another phenotype, CD45RA- PD1 + CD38 + -CXCR5-CD127-CD27 + , that when present on CD8 T cells is associated with transaminase levels, while when present on CD4 cells is linked to IgG levels [84]. You et al. reported that tissue resident CD8 T (CD8TRM) cells are elevated in the liver of patients with AIH compared to CHB, non-alcoholic fatty liver disease and healthy control tissues [85]. Their number correlated with biochemical and histological activity and decreased after glucocorticoid treatment [85]. Their potential pathogenic role warrants further evaluation. Schultheiß et al performed immuno-next generation sequencing on blood and liver tissue of patients with AIH, finding a TCR skewing—described as a biased signature of TRBV-J gene usage—in both peripheral and liver infiltrating T-cells of patients with AIH [86]. This skewing was unaffected by immunosuppressive treatment and unrelated to biochemical disease remission [86]. This suggested to the authors that steroid treatment acts on T-cell function rather than on the underlying pathological T-cell architecture in this disease, accounting for its high relapse rate [86]. The findings in these three recent publications will need to be externally evaluated and validated on well phenotyped populations of AIH patients.

Loss of tolerance towards self-antigens associated with regulatory T cell (Tregs) dysfunction is central to AIH pathogenesis [87]. Tregs constitute 5–10% of circulating CD4 T cells and express constitutively the surface marker CD25, which is the aforementioned IL-2 receptor subunit-α, and lack expression of CD127, the α chain of the IL-7 receptor. Their transcription signature is the transcription factor box P3 (FOXP3) [88]. Tregs play a central role in maintaining immune tolerance and have been extensively studied in AIH, providing solid evidence of functional and numerical impairment in this condition [87]. In patients with AIH-1 and AIH-2, Tregs are defective in number and this reduction is more evident at diagnosis and during relapses than during drug-induced remission [89]. Percentage of Tregs correlates inversely with markers of disease activity, i.e. anti-SLA and anti-LKM1 autoantibody titers, suggesting that impaired Treg numbers favor liver centered autoimmunity [90]. In addition, Tregs from AIH patients at diagnosis are less capable to control CD4 and CD8 effector cell proliferation compared to Tregs isolated from AIH patients during disease remission or from healthy subjects [91–94]. Moreover, effector CD4 T cells in AIH are less susceptible to Treg restraints, this defect being due to reduced expression of the receptor molecule Tim-3 (inhibitory receptor T-cell-immunoglobulin-and-mucindomain-containing-molecule-3), which through the binding of galectin-9 expressed by Tregs, induces effector cell death [89]. In AIH, CD39 Tregs (CD39 being a marker of highly active and suppressive Tregs) are reduced in number, do not hydrolyze adequately proinflammatory nucleotides and do not control efficiently IL-17 production by effector T cells. CD39 Tregs exhibit plasticity and become unstable in an inflammatory milieu, suggesting that impaired immunoregulation in AIH results not only from impaired Treg number and function, but also from transition of Tregs into effector cells [95].

A reported increase of liver tissue FOXP3 positive cells in AIH, in particular when the disease is active, has been interpreted as homing of Tregs in the target tissue. However, these studies were based on the expression of FOXP3 by the infiltrating lymphocytes. As this molecule is also associated with activation of CD4 cells—including effector cells [96]—, functional demonstration of regulatory properties would be needed to define their nature.

Restoration of Treg number and function could provide effective treatment for AIH. However, further confirmatory data are warranted, since it would be essential to devise strategies that prevent Tregs to convert into damaging effector cells within the inflammatory milieu [78, 97].

Intestinal microbiome may also be involved in the pathogenesis of AIH. Alterations in the composition of the intestinal microbiome (dysbiosis) were described in experimental autoimmune hepatitis [98].

A novel AIH animal model based on the nonobese-diabetic mouse transgenic for HLA-DR3 and immunized with a DNA plasmid coding for a fusion protein of P4502D6/FTCD, showed reduced diversity of gut bacteria as compared to wild type nonobese-diabetic mice immunized with the same antigen [98]. In view of the reciprocal influence between gut microbiome and adaptive immunity it is conceivable that shaping of the microbiome due to HLA-DR3 possession is involved in the development of liver centered autoimmunity. Compared to healthy volunteers, untreated AIH patients have impaired integrity of intestinal tight junctions; increased plasma lipopolysaccharide levels; and decreased number of intestinal anaerobes [99]. A study from China including 119 corticosteroid-naïve AIH-1 patients found, by analyzing fecal samples, a reduced bacterial diversity in AIH patients as compared to controls, and an enrichment of Veilonella dispar, correlating with transaminase levels [100]. Moreover, a gut microbial disease signature, potentially useful as an AIH biomarker, was identified [100]. The role of the microbiome in AIH needs to be further investigated both in humans and in animal models.

Data on the role of innate immunity in AIH are scanty. Natural killer cells type II, which are innate-like T cells recognizing lipid antigens, have been found to produce proinflammatory cytokines in AIH patients, in contrast to healthy controls [101]. A harming role for macrophages is suggested by the observation of increased expression of VAV1, a protein playing a role in T- and B-cell development and activation, by liver Kupffer cells in AIH patients, as well as by higher serum levels of CD163, produced by activated macrophages, which normalize during disease remission [102, 103].

### Animal models

Altough none of the available AIH animal models faithfully reflects human disease in all its features, they are helpful to investigate single pathophysiological steps of AIH. Particularly, none of the available models reproduces the chronic-relapsing course of AIH, leading to fibrosis and cirrhosis [104].

Before the identification of the liver autoantigens involved in the pathophysiology of AIH, mouse models based on immunization with liver extracts were established and widely used [105, 106]. These models have the advantage of being simple, but lack of knowledge about specific autoantigens hampers the detailed investigation of autoantigen-specific T cells. The first such model was established by Meyer zum Büschenfelde in 1972, who immunized rabbits with a human preparation containing two liver-specific proteins, one of which was later identified as the asialoglycoprotein receptor (ASGPR), a liver-specific protein expressed on the hepatocyte surface [106]. Animals developed liver damage with interface hepatitis as well as antibodies to ASGPR, which, however, failed to induce liver damage in transfer experiments [106]. In 1983, a transient liver damage was induced by transfer of spleen cells into naïve recipients using a similar mouse model by Kuriki et al. [107]. Later, a model based on immunization with the 100,000 g supernatant of syngenic liver homogenate, was published, leading to the in vitro identification of S-100-specific T cells [108, 109]. According to this model, p38 mitogen-activated protein kinase and nuclear factor kappa B play a role in the immunopathogenesis of experimental AIH [110]. However, the liver histology of the S-100 model does not show the typical AIH changes of interface hepatitis or centrilobular necrosis, and shows granulomas, which are not a feature of AIH [109].

Concanavalin A-induced hepatitis has been exensively used to investigate AIH pathophysiology [111]. Conacavalin A, a lectin extracted from jack-bean, is a T cell mitogen, leading to acute severe hepatitis with cytokine storm after systemic application and is therefore more a model of non-specific, T-cell mediated acute liver injury than of AIH [105]. Despite these limitations, it has provided evidence that Th1 cells and their cytokines IFNγ and TNFα can play a central role in inducing liver damage. Based on this model, it has also been shown that NKT cells, which secrete both IL-4 and IFNγ, are critical to the development of liver injury [112]. Moreover, a pathogenic role for IL-17C, produced by hepatocytes, and acting by binding to its cognate receptor expressed on liver resident T cells, has been shown in this model [113].

Subsequently, a variety of transgenic mice developing spontaneous AIH-like liver injury have been developed. Among these, a complex model combining PD-1 deficiency with neonatal thymectomy, leads to a fatal AIH-like hepatitis with ANA-positivity, suggesting a prominent protective role of Tregs in AIH, confirmed by reversion of progression to fatal hepatitis by adoptive transfer of Treg [114]. A model generated by triple knock-out of Tyro3, Axl and Mer, resulting in an excessive toll-like receptor (TLR)-dependent activation, suggests a prominent role of innate immunity in breaking tolerance to the liver [115]. A role of innate immunity is shown also by the model based on liver expression of IL-12, a Th1 differentiation signal cytokine: these mice expressing IL-12 under the control of a liver-specific promoter exhibit AIH-1-like disease, with ANA and SMA positivity, ipergammaglobulinemia, persistent mononuclear cell infiltration and fibrosis, as well as response to immunosuppressive drugs [116].

Based on the presence of AIH-2 in some 20% of APECED-syndrome patients, a transgenic model was generated by Hardtke-Wolenski et al., in which the AIRE1 gene is truncated at exon 2: 24% of these mice developed AIH-like liver damage [117].

An interesting AIH model has been generated by Bonito et al. based on depletion of medullary thymic epithelial cells, which ectopically express self-antigens in the thymus, leading to the elimination of autoreactive T cells [118]. Surprisingly, these animals do not show multiorgan autoimmunity, but develop an AIH-1 like disease, with interface hepatitis, positive ANA and anti-SLA antibodies, suggesting that intact central tolerance is key to prevent AIH-1 [118].

Another approach to generate transgenic AIH animal models is the expression of antigens under the control of liver-specific promoters: since this approach is not capable of inducing liver damage on its own in the liver, a tolerogenic organ, it has to be coupled with adjuvants and/or adoptive transfer of antigen-specific T cells. An example of such a model is the one generated by expression of ovalbumin under the control of the hepatocyte transferrin promoter coupled with transfer of ovalbumin-specific T cells [119]. These mice exhibit only transient hepatitis, though the main advantage of this model is the precise definition of the antigen and its liver restriction. Identification of the key autoantigen in AIH-2 has led to the establishment of models with liver expression of human CYP2D6, delivered by an adenovirus (Ad-2D6) [105], leading to persistent AIH-like disease with interface hepatitis, and liver infiltration of CD4 and CD8 T cells, B cells, as well as neutrophils, macrophages and dendritic cells, and positive anti-LKM1 antibody [120–122]. At variance with models using non-naturally occurring autoantigens, the CYP2D6 mouse develops a persistent hepatitis [104]. Interestingly, injection of human CYP2D6 does not cause AIH-like disease, demonstrating that the liver inflammation elicited by the adenovirus is instrumental to initiate the chronic autoimmune attack [105]. Similarly, immunization of non-obese diabetic mice with an adenovirus carrying FTCD leads to chronic AIH-like disease with liver fibrosis [123].

Another AIH-2 model uses repeated immunizations of C57BL/6 female mice with a plasmid encoding the antigenic region of human CYP2D6 and FTCD, together with the murine end terminal region of cytotoxic T lymphocyte antigen 4 (CTLA-4) as well as IL-12 to achieve tolerance breackdown [124]. This methodology leads to production of antigen specific autoantibodies, a relatively modest elevation of transaminase levels, and a portal/periportal inflammatory infiltrate composed of CD4 and CD8 T cells and, to a lesser extent, B cells. Using the same animal model, it was shown that adoptive transfer of ex vivo expanded CXCR3-positive Tregs induces disease remission [125]. Moreover, low dose anti-CD3 or anti-CD20 monoclonal antibodies substantially ameliorate liver damage, indicating the involvement of both T and B cells in producing liver injury [126, 127].

The genetic susceptibility to AIH is well demonstrated by an HLA-DR3 transgenic mouse on the non-obese diabetic background, which, upon immunization with a DNA plasmid coding human CYP2D6/FTCD fusion protein, develops ANA, anti-LKM1, anti-LC1, chronic immune cell infiltration of the liver parenchyma and fibrosis [98]. Interestingly, the same approach using an HLA-DR4 trangenic mouse showed less severe liver injury, reminiscent of less severe AIH seen in patients carrying the HLA-DR4 as compared to those carrying the HLA-DR3 allele [128].

## Clinical features and diagnosis

### Clinical presentation: adults

The clinical presentation of AIH in adults is very heterogeneous, ranging from asymptomatic cases to acute liver failure. The proportion of asymptomatic patients varies between studies from one in six to one in three. They are identified when liver function tests are performed for check-ups or insurance purposes. These patients have similar liver histology to symptomatic subjects, and need to be treated in order to avoid disease progression [129]. The most common clinical presentation is one of mild non-specific symptoms, including fatigue, arthralgias, malaise, anorexia, weight loss. In young females, amenorrhea is a typical presenting symptom; AIH can present during or shortly after pregnancy [130]. Extrahepatic autoimmune diseases affect 20–50% of AIH patients, and may be the leading clinical manifestation at diagnosis, autoimmune thyroid disease being the most common one.

About one third of AIH patients present acutely with jaundice, severe fatigue, nausea, and abdominal pain, meeting the criteria of acute severe AIH, which is defined by jaundice and INR between 1.5 and 2 in absence of known pre-existing liver disease. Very rarely, fulminant liver failure, defined by jaundice, INR ≥ 2 and hepatic encephalopathy occurring within eight weeks from illness onset in the absence of known pre-existing liver disease, is the initial presentation of AIH [1, 129]. These clinical pictures may be due to new onset AIH or to an acute exacerbation of pre-existing undiagnosed AIH, liver histology being key in differentiating between them, presence of advanced fibrosis/cirrhosis being suggestive of the latter. In fulminant liver failure, massive hepatic necrosis is typically seen. Acute deterioration due to superimposed viral infection, DILI or toxic causes needs to be accurately ruled out by taking a thorough clinical history and appropriate laboratory tests including polymerase chain reaction for hepatitis B, C and E viruses. Of note, autoantibodies may be negative and IgG may be normal in patients presenting acutely, becoming detectable after initiation of immunosuppression [1], though a recent study from China did find significantly higher IgG serum levels and ANA titers in acutely presenting patients [131]. A minority of patients present with established liver cirrhosis and complications of portal hypertension; histological liver inflammation may be absent (‘burnt-out cirrhosis’), the diagnosis relying on history, presence of extrahepatic autoimmune diseases and circulating autoantibodies [132].

One third of adult patients have cirrhosis at diagnosis, which has been associated by most, but not all, studies, with lower overall survival [129, 132, 133]. Cirrhosis stage is likely to play a prognostic role: according to a recent paper from India including 92 AIH patients aged >14 years, presence of severe ascites at diagnosis was associated with a 12-month transplant-free survival of only 25%, as compared to 96% in patients with compensated cirrhosis [134].

### Clinical presentation: children

Two thirds of pediatric AIH cases are AIH-1, which typically presents during adolescence, whereas AIH-2 affects younger children, including infants [135, 136].

The same female preponderance seen in adults is encountered in children. However, acute onset is more common in children, being the presenting clinical picture in up to 67% of the cases [136]. Fulminant presentation is more frequent in AIH-2, affecting up to one quarter of the cases; some 40% of AIH-1 children and 25% of AIH-2 children present mild, non-specific symptoms, similarly to adults [135]. More rarely, children present with signs and symptoms of cirrhosis and portal hypertension. Asymptomatic presentation is reported to be rare, but according to our experience, it is not infrequent, probably depending on local practice of performing blood tests in asymptomatic children, e.g. before minor surgery. According to a recent publication describing the long-term follow-up of 83 children with autoimmune liver disease, 20% of those with AIH were asymptomatic at diagnosis, possibly reflecting earlier diagnosis owing to increased disease awareness [136].

Children with AIH-2 are more frequently affected by concomitant autoimmune skin manifestations as compared to adults, mostly by vitiligo, alopecia, cutaneous vasculitis and urticaria [137, 138]. Partial IgA deficiency is seen in 40% of AIH-2 patients, in our experience not associated with an increased risk of respiratory infections [135]. Up to half of the AIH children have cirrhosis at diagnosis, with a lower proportion in more recent series, again suggesting an improved disease awareness and earlier diagnosis [19, 129, 132, 136]. Similarly to adults, cirrhosis at presentation has been associated with worse outcomes in some but not all series [136].

### Diagnosis

There is no single diagnostic test for AIH and diagnosis is based on a combination of clinical, biochemical, immunological and histological indices, and the exclusion of other known causes of liver disease that may share serological and histological features with AIH (e.g. hepatitis B, C and E, Wilson disease particularly in young patients, non-alcoholic steatohepatitis and DILI). A liver biopsy is mandatory in the diagnostic work up of AIH. DILI resembling AIH can be very difficult to differentiate clinically and histologically from classical AIH, the two main distinguishing features being lack of fibrosis/cirrhosis and ability to stop successfully steroid treatment after six months in DILI [132].

Diagnostic scoring systems have been developed by the IAIHG for adult patients [9, 139] where negative criteria (i.e. exclusion of viral hepatitides, Wilson disease or alcoholic liver disease, among others), are taken into account in addition to the positive criteria mentioned above to achieve a score of probable or definite AIH. The original (1993) and revised (1999) IAIHG scoring systems were devised mainly for research purposes to allow comparison between series from different centers, but have also been used clinically. Later the IAIHG published a simplified scoring system based on autoantibodies, IgG, histology, and exclusion of viral hepatitis that is better suited to clinical application [140].

However, the revised scoring system maintains a superior diagnostic performance [141], particularly for patients with comorbidities and alcohol or medication use, as confirmed by a comparative study by Gatselis et al. [142]. Therefore, patients with suspected AIH not reaching a diagnostic score result with the simplified scoring system, should be reassessed using the revised scoring system [71].

In the setting of severe acute AIH the simplified IAIHG scoring system has a low diagnostic performance, as patients may have normal IgG levels and negative autoantibodies [143]. In children, the IAIHG original, revised and simplified diagnostic scores are not suitable, since they do not allow to discriminate between AIH and ASC; furthermore, they do not consider the lower cut-off values of autoantibody titers significant in pediatrics [135]. Therefore, the European Society of Pediatric Gastroenterology, Hepatology and Nutrition (ESPGHAN) has issued in 2018 a pediatric diagnostic scoring system, which includes cholangiography, cut-offs of autoantibody titers adjusted to pediatric age, and measurement of peripheral anti-nuclear neutrophil antibody (pANNA) [135]. The score has been validated in a large King’s College Hospital cohort [69].

EASL and German-speaking countries guidelines recommend that every child diagnosed with AIH undergo a cholangiogram in order to rule out ASC [132, 144]. Cholangiography is not included in the AASLD guidelines [129].

### Autoantibodies

**Fig. 2:**
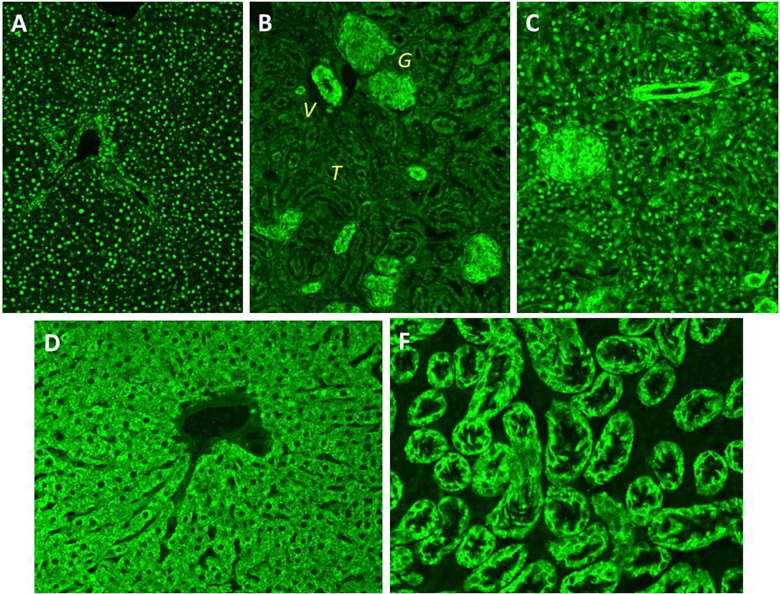
Autoantibodies detected by indirect immunofluorescence on rodent liver tissue. Autoimmune hepatitis type 1: Panel **A**: anti-nuclear antibody (ANA) homogenous pattern on liver tissue. Panel **B**: anti-smooth muscle antibody (SMA) on kidney tissue showing staining of vessels (V), glomeruli (G) and tubules (T), VGT pattern. Panel **C**: combined ANA and SMA patterns. Autoimmune hepatitis type 2: anti-liver-kidney microsomal type 1 (LKM-1) antibody pattern on liver (**D**) and kidney (**E**) tissue

A key diagnostic criterion for all AIH scoring systems is the detection of autoantibodies [9, 139, 140], which not only assists in the diagnosis but also allows differentiation of AIH types (Table 2). ANA and SMA characterize AIH-1, while anti-LKM-1 and anti-LC-1 define AIH-2, though occasionally ANA or SMA can coexist with anti-LKM-1 or anti-LC-1, the clinical course in these cases being similar to that of AIH-2. A major target of SMA is the actin of smooth muscle, whereas the molecular target of LKM-1 is CYP2D6 [145] and of anti-LC-1 is FTCD [146]. In the IAIHG scoring systems extra points are allocated to higher titers of ANA, SMA, anti-LKM-1 and anti-LC-1 as measured by indirect immunofluorescence (IIF) using rodent stomach, kidney and liver as substrate (Fig. 2) [9, 139, 140]. Other techniques, e.g. commercially available enzyme linked immunosorbent assays (ELISAs), often used when expertise for IIF is lacking, remain to be fully validated [147]. As IIF on rodent tissues is not universally available, an update of the simplified IAIHG criteria has been proposed recently by a European study aiming at evaluating the performance of a score including ANA tested on HEp2 cells and SMA tested by ELISA in adult patients [48]. The authors report a comparable sensitivity and specificity for ANA tested by IIF on tissue sections or on HEp2 cells, using the traditional cut-off of 1:40 for the former and a new cut-off of 1:160 for the latter [48]. The area under the curve (AUC) of an ELISA including nuclear HEp2 cells extracts as an antigenic source was better than an ELISA including a selection of nuclear autoantigens. Worryingly, the best performing ANA ELISA contained amongst its ‘nuclear’ target antigens also the mitochondrial M2 antigen, questioning the validity of the assay in the detection of ANA. It therefore comes as no surprise that the median values obtained with this ‘ANA test’ are significantly higher in PBC patients compared to AIH patients (49.6 Units in AIH vs. 161.7 Units in PBC), suggesting detection of mitochondrial in addition to nuclear reactivities. Last, in the proposed updated diagnostic criteria, the score of 6 for probable, and 7 for definite AIH can be achieved with 2 points awarded to strong positivity for ANA or SMA. However, the ELISA cutoff value for strong positivity is not standardized and needs to be established by each individual center, making the simplification of the simplified criteria a rather complicated matter.

**Table 2:** Diagnostic autoantibodies in autoimmune hepatitis

Autoantibody	Method of detection	Target antigen	Frequency in AIH		Clinical significance in AIH	Positivity in other diseases	Comments
			AIH-1	AIH-2			
ANA	IIF	Unknown in 1/3 of AIH patients. Histones, centromere, chromatin, double- and single-stranded DNA, cyclin-A and ribonucleoproteins	75%	Rare	Typical of AIH-1, coexisting with anti-SMA in 50% of AIH-1,	May be positive in a host of hepatic and extrahepatic diseases	Homogeneous IIF pattern in 2/3 of the cases; speckled in 1/3 of the cases. Multiple nuclear dots and rim-like IIF patterns are PBC-specific
SMA	IIF	Unknown in 20% Filamentous actin Desmin Vimentin	85–95%	Rare	Typical of AIH-1 at high titers; VG and VGT patterns are AIH-1 specific;	May be positive in a host of hepatic and extrahepatic diseases, particularly at low-titer and with V pattern	Titers correlate with disease activity
Anti-actin	Molecular assays	Filamentous actin	75%	Unknown	Typical for AIH-1 at high titers Usually coexists with SMA in AIH1	May be positive in a host of hepatic and extrahepatic diseases, particularly at low-titer	Sensitivity and specificity depend on the chosen cut off
Anti-LKM1	IIF Molecular assays	Cytochrome P4502D6 (CYP2D6)	Absent	Up to 90%	Diagnostic of AIH-2 in absence of HCV infection	HCV (up to 11%) Very rare in ASC	Titers correlate with disease activity;
Anti-LC1	IIF (masked in the presence of concomitant anti-LKM) Molecular assays	formiminotransferase cyclodeaminase	Very rare	Up to 60%	Diagnostic of AIH-2 in absence of HCV infection; Combined with anti-LKM1 in 50% of the cases	HCV (rare) ASC	Titers correlate with disease activity
Anti-SLA	Molecular assays	O-phosphoseryl-tRNA:selenocysteine-tRNA synthase (SEPSECS)	20–30%	20–30%	Highly specific (98.9%)	HCV (very rare)	Present in up to 58% in AIH-1 and AIH-2 if tested by radioligand assays. Prognostic for aggressive disease
pANNA	IIF	Unknown	50–96%	Absent	May be the only serological marker in AIH-1	IBD PSC ASC	

*ANA* antinuclear-antibody, *AIH* autoimmune hepatitis, *IIF* indirect immunofluorescence, *SMA* smooth-muscle antibody, *V* vessel, *VG* vessel glomerular, *VGT* vessel glomerular tubular, *LKM* liver kidney microsomal, *LC* liver microsomal, *SLA* soluble liver antigen, *pANNA* peripheral anti-nuclear neutrophil antibodies, *IBD* inflammatory bowel disease, *ASC* autoimmune sclerosing cholangitis, *PSC* primary sclerosing cholangitis

Anti-LC-1 can be present on its own, but frequently occurs in association with anti-LKM-1. This co-occurrence can go unnoticed because anti-LKM-1 obscures the anti-LC-1 pattern. Anti-LC-1 can also be detected by commercial tests (ELISAs, line blots and immunoblots). Positivity for autoantibodies is not sufficient for the diagnosis of AIH since they can be present, usually at low titer, in other liver disorders such as viral hepatitides [148], Wilson disease [149] and non-alcoholic steatohepatitis [150]. Other autoantibodies less commonly tested but of diagnostic importance include peripheral anti-nuclear neutrophil antibody (atypical pANCA or pANNA or NANA) and anti-SLA. pANNA is frequently found in AIH-1 and in ASC, and is also common in IBD, while it is virtually absent in AIH-2. Anti-SLA, originally described as the hallmark of a third type of AIH [151], is also found in up to 50% of patients with AIH-1, AIH-2 or ASC, where it defines a more severe course [152]. Anti-SLA is not detectable by conventional immunofluorescence, but the definition of its molecular target as SEPSECS [153] has enabled the establishment of molecularly based diagnostic assays. Anti-SLA is highly specific for AIH, but currently available immunoassays have low sensitivity. There is a small proportion of patients with AIH without detectable autoantibodies. This condition, which responds to immunosuppression like the sero-positive form, represents sero-negative AIH [154].

### Liver histology

**Fig. 3:**
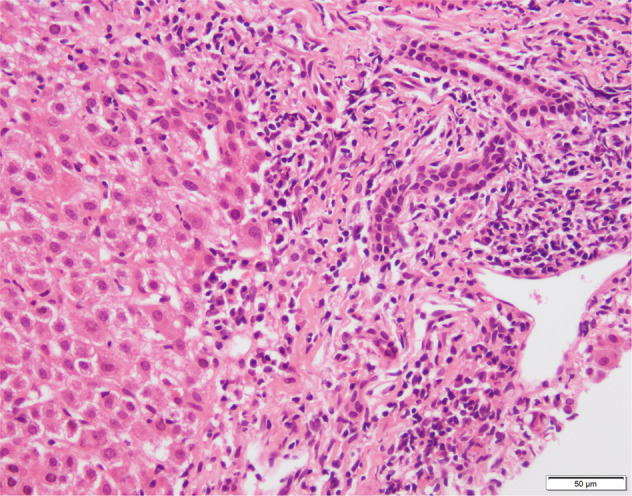
Interface hepatitis in a patient with autoimmune hepatitis type 1. Lymphocytes and plasma cells infiltrate the portal and periportal area, extending to and disrupting the parenchymal limiting plate. Hematoxylin & eosin staining; original magnification x100. Courtesy of Professor Yoh Zen, Institute of Liver Studies, King’s College Hospital, London, UK

Liver biopsy is necessary to establish the diagnosis of AIH, the typical histological picture being a dense mononuclear and plasma cell infiltration of the portal areas, which expands into the liver lobule leading to damage of the hepatocytes at its periphery with erosion of the limiting plate (‘interface hepatitis’) (Fig. 3). Hepatocytes surrounded by inflammatory cells become swollen and undergo pyknotic necrosis. Plasma cells are usually abundant at the interface and within the lobule, but even their presence in low number is compatible with the diagnosis of AIH. When AIH presents acutely or at the time of relapse, panlobular hepatitis with connective tissue collapse resulting from hepatocyte death and expanding from the portal area into the lobule (‘bridging collapse’) is often observed. Non-specific features that may point to the diagnosis of AIH are emperipolesis and hepatocyte resetting [155]. The typical histological picture of interface hepatitis might not always be present at diagnosis, as it varies according to disease stage or previous immunosuppressive treatment for associated conditions [156]. It has been suggested that in pediatric AIH hyaline droplets in Kupffer cells might be a useful diagnostic marker to distinguish AIH from other forms of chronic hepatitis, the hyaline droplets being positive for IgG by immunohistochemistry and correlating with a > 2-fold increase in serum level of IgG [157].

Histology also allows evaluating the extent of fibrosis and helps in identifying overlap syndromes or possible presence of concomitant diseases, such as non-alcoholic fatty liver disease [158]. Though inflammatory changes surrounding the bile ducts have been reported also in a proportion of patients with classical AIH [159], when conspicuous they suggest an overlap with sclerosing cholangitis.

### Non-invasive fibrosis assessment

Similarly to other liver diseases, presence of advanced fibrosis is associated with worse outcome in AIH [160]. Non-invasive fibrosis assessment is therefore highly relevant in patients’ management. Vibration-controlled transient elastography (VCTE) has entered clinical routine in hepatology, and its accuracy has been evaluated also in AIH. Liver stiffness is increased in the presence of histological inflammation, leading to the recommendation of deferring its use in AIH patients until remission has been achieved. A recent study reported that spleen stiffness measurement is less influenced by liver inflammation and can facilitate fibrosis assessment in untreated AIH patients [161] The hepatic cut-off with the highest sensitivity and specificity for advanced fibrosis in AIH is 10.5 kPa. Moreover, VCTE has been reported to be a valuable tool in monitoring fibrosis regression in AIH patients achieving biochemical remission [162].

### Treatment

**Fig. 4:**
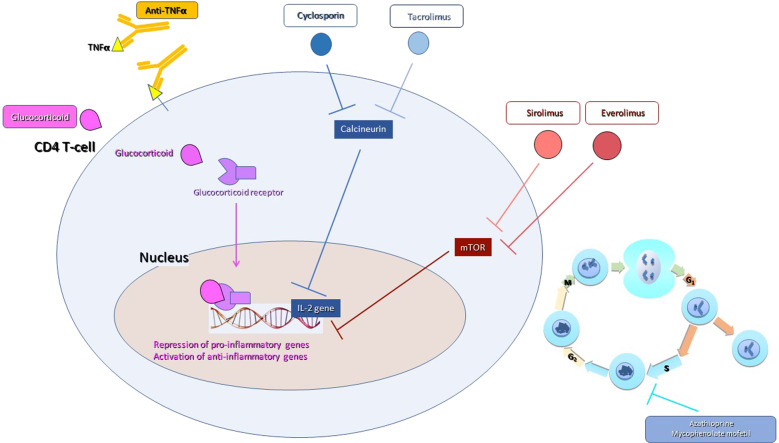
Overview of the mode of action of initial and second/third-line pharmacological treatments used in autoimmune hepatitis. Glucocortisteroids bind to the cytosolic glucocorticosteroid receptor, which migrates to the cell nucleus leading to repression of pro-inflammatory genes and activation of anti-inflammatory genes. Azathioprine and mycophenolate mofetil inhibit the synthesis of purines, the substrates for RNA and DNA synthesis during the S phase of the cell cycle, thus causing cell death of the rapidly dividing cells, including lymphocytes. Calcineurin inhibitors act mainly by suppressing the synthesis of IL-2, which is essential to T cell proliferation. Sirolimus and everolimus act on the mammalian target of rapamycin (mTOR), a serine/threonine-specific protein regulating cellular metabolism, growth, and proliferation. Anti-tumor necrosis factor (TNFα) antibodies act by binding to soluble or membrane-bound TNFα preventing its binding to the cognate receptor

The aim of treatment is to achieve biochemical remission, defined as normal serum transaminase and IgG levels; in children, negative or low-titer autoantibodies are also part of the definition of remission, since it has been shown that anti-LKM1, anti-LC1 and SMA titers correlate with disease activity in this age group [2, 129, 132]. Biochemical remission parallels improvement of histological activity and its maintenance prevents disease progression [163]; AIH-related symptoms also disappear on biochemical remission [129, 132, 135]. Conversely, failure to achieve biochemical remission, either due to intolerance or insufficient response to treatment, leads to histological progression, requiring alternative therapeutic approaches, mostly using off-label drugs [129, 132, 135]. Fig. 4 summarizes the mode of action of initial and second/third-line immunosuppressants used in AIH.

In AIH patients affected by Covid-19, immunosuppressive treatment has not been associated with worse outcomes [164, 165].

### Standard treatment

Standard treatment includes predniso(lo)ne and azathioprine, and is effective in 80–90% of the patients [80].

Corticosteroids are the backbone of AIH treatment, in both children and adults; they are very effective in the vast majority of patients in achieving biochemical remission. Absence of transaminase level decrease on steroids questions the diagnosis of AIH [129, 132, 135]. The survival benefit of treatment is demonstrated by placebo-controlled trials from the ‘70 s, reporting a mortality as high as 56% during a 30–72 months follow-up period in untreated patients, as compared to 14% in treated patients [166–168].

Glucocorticoids act by binding to their cognate receptor, the glucocorticoid receptor (GR), leading to induction or repression of thousands of genes. Their anti-inflammatory effect is mediated by T cell signaling and downregulation of proinflammatory cytokine production [169]. Moreover, they stimulate the proliferation of Tregs [170].

Predniso(lo)ne is the steroid of choice in AIH. In adults, the EASL guidelines recommend an initial dose ranging from 0.5 to 1 mg/kg/day, thus leaving the clinician to choose the most appropriate dose for each patient. The AASLD guidelines recommend starting predniso(lo)ne treatment with 60 mg/day in acute severe cases and with 20–40 mg/day in all other cases, acute severe AIH being defined as presence of jaundice, INR > 1.5 < 2 in absence of encephalopathy and of previously recognized liver disease [129, 132]. Chinese adult AIH guidelines recommend an initial predniso(lo)ne dose of 40–60 mg/day if used alone, and 30–40 mg if used in combination with azathioprine [130]. In children, the recommended initial predniso(lo)ne dose is 2 mg/kg/day (maximum dose 60 mg/day). Predniso(lo)ne dose should be tapered on a weekly basis under strict transaminase control; if transaminase levels stop decreasing or increase, azathioprine should be added at a starting dose of 0.5 mg/kg/day to be increased weekly to a maintenance dose able to maintain normal transaminase levels (1.5–2 mg/kg/day). Ultimately, 85% of children will need azathioprine. It should be stressed that treatment must be tailored to the single patient, taking into account disease severity, age, drug tolerance, comorbidities and response. Therefore, clinicians should abstain from applying proposed schedules indistinctly to every patient. A rapid decline of serum transaminase levels, defined as 80% drop within the first 8 treatment weeks, predicts transaminase normalization at 26 and 52 weeks [171].

The appropriate initial predniso(lo)ne dose has been debated. Schramm et al. reported in 2010 a cohort of 92 adult patients treated with an initial predniso(lo)ne dose of 1 mg/kg/day plus azathioprine in non-jaundiced patients; this regimen was associated with faster achievement of biochemical remission, and less steroids side effects as compared to standard regimens [163]. Recently, a multicenter retrospective European study reported a similar frequency of normal transaminase levels after six months of therapy in adult AIH patients treated with high (≥0.5 mg/kg/day) or low (<0.5 mg/kg/day) predniso(lo)ne doses; however, patients in the high dose group had higher median ALT and bilirubin levels, suggesting that the initial dose should be adapted to disease severity [172]. Steroid adverse effects after one year of treatment were similar in the two groups [172].

Rapid predniso(lo)ne dose tapering under strict transaminase control is key to minimize side effects and therefore maximize adherence. Since the GRs are pleiotropically expressed, steroids have systemic side effects of various severity, including weight gain and Cushingoid aspect, insomnia, osteopenia/osteoporosis, hyperglycemia, and, less commonly, brittle diabetes, hypertension, psychosis, cataract and glaucoma, increased risk of infections, hypertrichosis and acne. Even non-severe side effects may have a negative impact on the quality of life of the patients, jeopardizing adherence. Side effects are associated with high doses and long-term exposure. Recently, it has been reported that even low-dose prednis(ol)one (≤5 mg/day) increases the risk of bone fractures, whereas diabetes and cataract were associated with higher doses [173]. The benefit of weekly blood tests enabling swift steroid reduction outweighs the discomfort of this strategy, which should be carefully explained to the patients and their families.

As mentioned above, azathioprine is the first-choice steroid sparing agent in AIH, and is part of the standard treatment. It has been used since the ‘70 s, being one of the few available immunosuppressive drugs at that time. It is an antagonist of purine metabolism, inhibiting RNA and DNA synthesis, and therefore affecting the more rapidly dividing cells including lymphocytes. Azathioprine should be added after some two weeks of steroid treatment, since it can be hepatotoxic, particularly in jaundiced patients; it should be started at low dose (50 mg/day in adults, 0.5 mg/kg/day in children) and increased gradually up to 150 mg/day in adults, and 1.5–2 mg/kg/day in children, if tolerated, under monitoring of the blood cell count for its potential myelotoxicity [129, 135]. Delayed azathioprine initiation allows also differentiating azathioprine hepatotoxicity from steroid non-response. Mild nausea is a common side effect, which can be mitigated by splitting the dose during the day and taking the drug after meals; however, some patients develop severe nausea and vomiting, requiring drug discontinuation. A recent large international retrospective study reported azathioprine discontinuation in 15% of the patients during the first year of treatment for side effects, mostly gastrointestinal [174]. The same paper reports hepatotoxicity in some 2% of the patients, irrespective of initiation simultaneously to steroids or two weeks later [174]. Azathioprine hypersensitivity syndrome is characterized by systemic symptoms including fever, myalgia, rash, arthralgia and nausea, developing in the first days/few weeks after starting treatment; rechallenge should be avoided. Its frequency varies across series, being as high as 9% in a recent series of adult patients with ANCA-associated vasculitis and concomitant steroid treatment, and 5% in the only AIH series published [175, 176]. Cutaneous squamous cell carcinoma is an additional reported side effect of long-term azathioprine exposure [177, 178]. Besides being dose-dependent, azathioprine myelotoxicity is influenced by genetic polymorphisms in the gene encoding the enzyme thiopurine methyltransferase (TPMT) which converts 6-mercaptopurine (6-MP) to the toxic metabolite 6-methylmercaptopurine (6-MMP); 0.3% of individuals have very low or absent TPMT activity [179]. Testing for polymorphisms of the TPMT gene can be performed, if available, to avoid severe myelotoxicity in subjects with low/absent TPMT activity, but the majority of patients with azathioprine myelotoxicity have normal TMTP activity: therefore, close monitoring is mandatory in every patient [179]. Conversely, patients with low TMTP activity may tolerate the drug well [179].

Due to the high inter-individual variability of azathioprine metabolism, measurement of its metabolites 6-MMP and 6-TGN is useful in patients with insufficient response, not only to check adherence, but also to optimize treatment: if both levels are low, azathioprine dose can be increased under strict monitoring of the blood cell count. A skewed azathioprine metabolism is mirrored by high 6-MMP (usually > 5000 pmol/8 × 108 red blood cells) and low 6-TGN levels (usually < 75 pmol/8 × 108 red blood cells): in this case, allopurinol (100 mg/day in adults) should be added, and azathioprine dose reduced to 25–30%, under monitoring of 6-MMP and 6-TGN levels, followed by a gradual increase of the azathioprine dose in case of insufficient response [180]. Optimal 6-TGN levels in AIH are not defined: according to a retrospective study, and similarly to the target levels in IBD treatment, a reasonable target level is 220 pmol/8 × 108 red blood cells [179]. A recent retrospective study from King’s College Hospital, London, confirmed the benefit of monitoring azathioprine metabolites in AIH patients in terms of achievement of biochemical remission [181]. This study also showed that low 6-TGN levels (75–225 pmol/8 × 108 red blood cells) were sufficient to maintain biochemical remission in a high proportion of patients, who had fewer side effects compared to those with higher levels, again stressing the importance for treatment to be tailored to the single patient [181].

Azathioprine monotherapy at a dose of 2 mg/kg/day (up to 2.5 mg/kg/day in children) may be used to maintain biochemical remission, but in our experience a combination of low to medium doses of azathioprine with low-dose predniso(lo)ne is more effective for this purpose [136].

Azathioprine is safe in pregnancy. A recent large retrospective French study confirmed that azathioprine exposure during the first trimester is not associated with increased risk of birth defects, and exposure in the third trimester does not increase the risk of pre-term birth, which, when it occurs, is most likely due to the maternal disease [182]. Azathioprine treatment should not be discontinued during pregnancy as poor disease control is more dangerous than the very low risk of fetal side effects [183, 184].

Budesonide is a glucocorticoid whose pharmacokinetics makes it attractive as a treatment option for AIH, having >90% first pass liver uptake [22]. However, it is contraindicated in cirrhotic patients due to an increased risk of vascular complications [185]. Following the results of a randomized controlled trial showing higher transaminase normalization rate at 6 months with budesonide/azathioprine as compared to prednisone/azathioprine, budesonide has been approved for the initial AIH treatment in adults [186]. However, the trial has been criticized for its design: while prednisone dose was reduced per-protocol, budesonide was reduced according to the biochemical response. Moreover, the response rates in both treatment arms were lower than those obtained with the standard treatment described above, possibly because, besides treatment naïve patients, also relapsing, and thus difficult to treat, patients were included in the trial, and because initial prednisone doses were lower than those recommended by guidelines, particularly for pediatric patients [186]. Last, all patients were prescribed azathioprine from the beginning, making it impossible to differentiate azathioprine hepatotoxicity from non-response [186]. A sub-analysis of the 47 pediatric patients included in the trial (aged 9–17) did not show a significant difference in biochemical remission rates between the budesonide and the prednisone arms [187]. The remission rate in both arms was lower than the one reported with standard treatment (50% vs. 90%), and therefore budesonide cannot be recommended as initial AIH treatment in children and adolescents [188]. Budesonide has probably its place in the treatment of AIH in adult non-cirrhotic patients with steroid side effects on prednisone, rather than as first-line treatment for every patient [189]. However, steroid side effects occur also on budesonide, as shown by a recent retrospective study in which budesonide in AIH was associated with an increased risk of bone fractures and cataract in the long-term [173].

### Second-line treatment

Patients intolerant to or with an insufficient response to predniso(lo)ne/azathioprine need alternative treatments.

Azathioprine-intolerant patients can be switched to 6-mercaptopurine (6-MP), since it has been shown in small studies that 50–75% of these patients tolerate 6-MP [190, 191]. Better 6-MP tolerance in azathioprine-intolerant patients has been more robustly documented in IBD [192].

Another possible but less documented strategy for azathioprine-intolerant AIH patients is the use of 6-thioguanine, which is enzymatically converted to 6-TGN, bypassing the metabolic step leading to 6-MMP formation. Similarly to 6-MP, there is more experience in IBD treatment, only a small number of AIH patients having been reported to have been successfully switched to this compound [193]. High thioguanine doses have been associated with an increased risk of non-cirrhotic portal hypertension [194].

Mycophenolate mofetil (MMF), which inhibits purine synthesis in B and T lymphocytes, is used off-label in AIH patients intolerant to azathioprine and 6-MP, as well as in those with unsatisfactory response to standard treatment, being more effective in the latter group [129, 132]. It is usually started in adults at 500 mg twice daily and increased if tolerated to 1000 g twice daily; in children the starting dose is 5 mg/kg/twice daily, with a maximum dose of 20 mg/kg/twice daily [132, 135]. It is generally better tolerated than azathioprine, the most frequent side effect being gastrointestinal symptoms [195]. The main drawback of this compound is its teratogenicity, a serious problem since a large proportion of AIH patients are women of child bearing age [129, 132]. MMF has been reported to be effective also as first line AIH treatment in a large real-world study, but it has not been compared to standard treatment [196].

### Third-line therapy

Some 10–20% of AIH patients are difficult to threat and should be managed in referral centers. Often a combination of immunosuppressive drugs is needed. Randomized controlled trials are lacking, and recommendations are based on retrospective series and single center experiences.

Calcineurin inhibitors. Cyclosporin A has been used as a rescue therapy in adults with AIH, with limited data available in the literature, including small retrospective series, small prospective uncontrolled and open studies and case reports [197, 198]. Even more limited data have been published on calcineurin inhibitors as first line therapy in adults [198, 199]. In children, cyclosporin monotherapy has been used as induction treatment, first in a prospective multicenter uncontrolled trial, and later in a randomized study, performed by the same group, comparing cyclosporin with standard treatment, with similar results in both arms, except for earlier remission achievement with standard treatment [200, 201]. Toxicity included Cushigoid features with standard treatment and gingival hypertrophy with cyclosporin [202]. Cyclosporin has been used as second-line treatment for acute severe AIH in a small adult series from Japan, with good results [203]. In pediatrics, combined steroid/cyclosporin therapy has shown similar results to steroids alone in patients with acute presentation and prothrombin time <50% [204].

Tacrolimus has been used both as first-line and rescue therapy in children and adults with AIH. The quality of the available evidence is low. According to a recent retrospective multicenter study, tacrolimus was equally effective as MMF as second-line treatment in patients who are either intolerant or insufficient responders to standard first-line treatment [205]. In children, reported efficacy as first line treatment is disappointing, whereas as second-line option it showed encouraging results in a recent retrospective multicenter study [206]. Due to its toxicity, tacrolimus should only be considered as third-line treatment [207]. There is anecdotal experience of tacrolimus as rescue treatment in adults with acute severe AIH [208]. Mammalian target of rapamycin inhibitors (mTOR inhibitors). mTOR inhibitors control the proliferation and survival of activated lymphocytes. There are few reported cases of AIH patients unresponsive to standard drugs treated with mTOR inhibitors, with variable results; the side effects include hyperlipidemia, mouth ulcerations, legs ulcers, thrombocytopenia, proteinuria, rash, and decreased resistance to infection [209, 210].

Treatments targeting B lymphocytes. Data on efficacy and safety of Rituximab, a monoclonal chimeric anti-CD20 antibody, as rescue treatment for AIH derive mainly from a small open-label study and a recent series, both showing beneficial effects without safety concerns [211, 212]. Ianalumab, a monoclonal antibody targeting anti-B cell activating factor (BAFF) receptor is currently being tested in a large multicenter, randomized, double-blind, placebo-controlled phase 2–3 clinical trial in AIH patients with incomplete response or intolerance to standard treatment (NCT03217422). Ianalumab has shown good safety profile in a phase 2 trial in Sjögren syndrome [213]. BAFF is a cytokine promoting proliferation and differentiation of B cells, BAFF receptors being expressed on mature B cells, in contrast to CD20, which is expressed also in early stages of B-cell maturation. BAFF levels are elevated in AIH and decrease with corticosteroid treatment [214, 215]. Belimumab is another monoclonal antibody targeting BAFF and licenced for the treatment of lupus erythematosus. Recently, good experience in two refractory AIH cases was reported [216].

Anti-TNFα agents. These drugs are extensively used in IBD, dermatological and rheumatological diseases. Though infliximab has been reported to be effective in normalizing serum transaminase levels in 8/11 adult AIH patients, caution is required not only for infectious complications, but also for potential induction of DILI resembling AIH [217–219]. Physicians should be aware of this possible complication while caring for AIH patients on anti-TNFα drugs for concomitant extrahepatic autoimmune diseases.

Toll-like receptor 4 antagonist. TLR4 is a cell surface receptor belonging to the pattern recognition receptor family, which, upon ligand binding and intracellular signaling involving NF-kB and MAPK, leads to upregulation of the proinflammatory cytokines IL-1β, IL-6 and TNFα. The TLR4 antagonist JKB-122, following demonstration of anti-inflammatory and hepatoprotective properties in animal models of AIH, has entered a phase II clinical trial, whose results have not yet been published (NCT02556372).

Low-dose IL-2. Tregs constitutively express the heterotrimeric IL-2 receptor, i.e. the IL-2Rα (CD25), IL-2Rβ (CD122), and IL-2Rγc (CD132), in contrast to conventional T cells, which express only transiently the heterotrimeric form of IL-2. Low-dose IL-2 treatment may therefore shift the balance of the autoimmune response towards regulation. Following the report of two cases, an uncontrolled, open label phase I/IIa clinical trial of low dose IL-2 is ongoing (NCT01988506): preliminary results in two AIH patients showed short-term Treg expansion without safety issues [220].

### Treatment of acute-severe AIH

AIH presenting as severe acute disease or fulminant liver failure remain challenging and require early consideration for liver transplant [129, 132, 221]. In fulminant liver failure, defined by the presence of encephalopathy, a short trial of corticosteroids has rarely shown to be beneficial, and patients should undergo urgent liver transplantation [129, 132]. In absence of hepatic encephalopathy, a trial of corticosteroids 1 mg/kg/day is advisable, with assessment of response after 7 days and referral for transplant in absence of improvement of INR and bilirubin [129, 132, 221]. Improvement of MELD, UKELD and MELD-Na at day 7 has been used to help assessment of treatment response, without defined threshold values for these scores [222].

### Treatment withdrawal

Treatment should be continued for a minimum of three years, and for at least two years after achievement of biochemical remission [129, 132]. Histological activity is present in some 50% of treated AIH patients with normal transaminase levels, and is associated with an increased risk of relapse after treatment withdrawal [132, 223]. Therefore, a liver biopsy is advisable before treatment discontinuation, and treatment should be continued in presence of histological activity [80, 132]. Only some 20% of AIH-1 patients maintain remission off treatment, whereas in AIH-2 relapse is almost the rule [132, 135]. Treatment withdrawal should not be attempted just before or during puberty, when relapse is reportedly more common [135].

## Concluding remarks

Intriguingly autoimmunity in AIH is directed against a highly tolerogenic organ, the liver. AIH should be considered in the differential diagnosis of any instance of increased liver enzyme levels. Thus, disease awareness and timely diagnosis are crucial, since untreated disease has a poor prognosis. Several pathogenic aspects of AIH have been elucidated, including predisposing genetic factors and some disease-specific humoral and cellular immune responses. However, clear knowledge on initial triggers, immunopathogenic mechanisms and effector processes remain elusive. A better understanding of each of these aspects would facilitate the establishment of novel treatments aimed specifically at arresting liver autoaggression or, ideally, at reinstating failed tolerance to liver autoantigens, thereby abrogating our current reliance on non-specific immunosuppression with its attendant side effects.
